# Clinical practice insight: vasoactive-inotropic drugs do not impede early enteral nutrition in pediatric ECMO support

**DOI:** 10.3389/fnut.2025.1676280

**Published:** 2025-10-13

**Authors:** Ye Ren, Run Zhou, Hongxing Dang, Yueqiang Fu, Chengjun Liu, Jing Li

**Affiliations:** ^1^Ministry of Education Key Laboratory of Child Development and Disorders, Department of Pediatric Intensive Care Unit, Children’s Hospital of Chongqing Medical University, Chongqing, China; ^2^National Clinical Research Center for Child Health and Disorders, China International Science and Technology Cooperation Base of Child Development and Critical Disorders, Chongqing, China; ^3^Chongqing Key Laboratory of Child Health and Nutrition, Chongqing, China

**Keywords:** vasoactive-inotropic drugs, extracorporeal membrane oxygenation, early enteral nutrition, energy intake adequacy, pediatrics

## Abstract

**Objective:**

To evaluate whether vasoactive-inotropic drug use impedes the early initiation of enteral nutrition (EN) and affects energy intake adequacy in pediatric patients receiving extracorporeal membrane oxygenation (ECMO) support.

**Methods:**

A prospective observational cohort study was conducted among pediatric ECMO patients between June 2018 and June 2024. Patients were categorized into early (≤ 48 h) and delayed (> 48 h) EN initiation groups, and into energy-deficient (< 30% of energy target) and non-deficient (≥ 30%) groups based on daily EN energy intake during the first five ECMO days. Vasoactive-Inotropic Score (VIS), PRISM III score, EN interruptions, and energy intake adequacy were analyzed. Spearman correlation and Cohen’s d were used to explore associations between VIS and EN intake. A support vector machine (SVM) model was used to identify predictors of energy intake status.

**Results:**

A total of 64 patients were included, with 43 (67.2%) receiving EN within 48 h. VIS did not significantly differ between early and delayed EN groups. Delayed EN was associated with higher PRISM III scores (*P* = 0.037), lower EN energy intake (*P* < 0.001), and more frequent EN interruptions (*P* = 0.028). Among patients with EN intake <30% of the target, VIS was significantly higher (*P* < 0.05). VIS on days 1 and 2 were the top predictors in the SVM model (25.7% and 27.4%, respectively).

**Conclusion:**

Vasoactive-inotropic drug use does not impede the early initiation of EN in pediatric ECMO patients. However, higher VIS in the initial 48 h is associated with suboptimal energy delivery. These findings suggest that while EN can be started early despite vasoactive support, closer monitoring of hemodynamic status is warranted to optimize nutritional adequacy.

## 1 Introduction

Enteral nutrition (EN) is a cornerstone of supportive care in critically ill pediatric patients. Despite its established benefits, the incidence of malnutrition remains high in pediatric intensive care units (PICUs), particularly among children requiring extracorporeal membrane oxygenation (ECMO) support (1, 2). These patients frequently fail to meet their caloric and protein requirements, placing them at significant nutritional risk (3, 4). The ECMO circuit itself triggers immune activation, oxidative stress, and systemic inflammation, all of which increase energy demands and compound the risk of under nutrition (5). Prior studies suggest that achieving nutritional targets through EN may be associated with improved survival in pediatric ECMO patients (6).

Many children undergoing ECMO require vasoactive-inotropic support to stabilize hemodynamics in the context of reduced cardiac output, capillary leakage, or fluid loss, especially in the early stages of ECMO (7). These medications may further impair gastrointestinal perfusion and function, increasing the risk of multiple organ dysfunction and intestinal ischemia. Additionally, inadequate nutrient intake during ECMO support can impair intestinal function and compromise barrier integrity, leading to bacterial translocation or sepsis, which further exacerbates hemodynamic instability and increases mortality rates (8).

However, existing guidelines have not clearly defined the relationship between the use of vasoactive-inotropic drugs and the adequacy of EN in pediatric ECMO patients (9, 10). In 2022, the Extracorporeal Life Support Organization (ELSO) issued the first guidelines on nutritional support and assessment for neonates and children receiving ECMO (9). The guidelines recommend initiating EN as soon as possible within 48 h after clinical stabilization in all pediatric ECMO patients. This aligns with the European Society of Pediatric and Neonatal Intensive Care (ESPNIC) guidelines, which suggest that EN should be started early if gastrointestinal function permits, and that the use of vasoactive-inotropic drugs does not preclude the initiation of EN, but the level of evidence remains low (11). Therefore, further research is needed to understand the impact of vasoactive drug use on EN in pediatric ECMO patients.

This study aims to evaluate whether the use of vasoactive-inotropic drugs impedes the early initiation of EN and affects the adequacy of energy intake in pediatric ECMO patients. By examining this relationship in a clinical setting, we hope to provide practical evidence to guide nutritional strategies in critically ill children receiving ECMO support.

## 2 Materials and methods

### 2.1 Study participants

This study was a prospective observational cohort study conducted in two PICUs of the Children’s Hospital of Chongqing Medical University and the Chongqing Key Laboratory of Child Health and Nutrition. The study included pediatric patients who continuously received ECMO treatment between June 2018 and June 2024. The Children’s Hospital of Chongqing Medical University is a tertiary pediatric teaching hospital, and its two PICUs are national key clinical specialties in China. This study did not involve any changes to the treatment plans of the pediatric patients during its execution. Data were prospectively collected using standardized case report forms and cross-checked with the electronic medical record system. To ensure data quality, the dataset was reviewed by two independent investigators, and any inconsistencies were resolved by checking the source records, thereby minimizing the risk of bias due to missing data. All data were anonymized, and the observed parameters did not involve any patient privacy. The study was approved by the ethics committee, and informed consent was obtained from the pediatric patients and/or their guardians.

### 2.2 Inclusion and exclusion criteria

**Inclusion criteria:** (1) Pediatric patients receiving ECMO treatment in either V-A or V-V mode; (2) No contraindications to EN; (3) Age ≤18 years.

**Exclusion criteria:** (1) Presence of primary gastrointestinal disease causing gastrointestinal dysfunction prior to ECMO treatment; (2) Duration of ECMO treatment less than 72 h; (3) Unavailable or incomplete medical records.

### 2.3 Study variables and objectives

#### 2.3.1 Nutritional-related variables

Whether EN was initiated within 48 h during ECMO treatment, and daily energy and protein intake via EN during the first 5 days on ECMO.

#### 2.3.2 Vasoactive drug-related variables

Types and dosages of vasoactive-inotropic drugs, and calculation of the Vasoactive-Inotropic Score (VIS) before ECMO initiation and for each day during the first 5 days on ECMO.

#### 2.3.3 General information

Age, gender, weight-for-age z-score, PRISM III score, duration of mechanical ventilation, primary cause for initiating ECMO, ECMO mode and duration, ECMO flow rate, 28-day PICU-free days, total hospital length of stay, and survival status at discharge.

#### 2.3.4 Study objectives

To compare the characteristics and outcomes between early and non-early EN initiation, to assess clinical features related to EN energy deficiency and non-deficiency, and to identify factors associated with energy intake below 30% of EN targets.

### 2.4 Nutritional goals and related definitions

The nutritional therapy plan for each pediatric patient was jointly formulated by the PICU attending physician and the clinical nutritionist, strictly following the treatment protocols. The energy and protein intake targets for each patient were calculated based on the Schofield equation. A detailed assessment of the patient was conducted every 4 h, and EN was initiated immediately once it was confirmed that there were no contraindications to EN. Feeding tolerance was continuously evaluated after EN initiation. Feeding intolerance was defined as the occurrence of severe gastrointestinal complications, including necrotizing enterocolitis, gastrointestinal bleeding, stress ulcers, severe abdominal distension, diarrhea, or vomiting.

If no signs of feeding intolerance were observed, the EN infusion rate was advanced stepwise according to the target prescription (typically 1–2 ml/kg per step). If intolerance was identified, EN was paused and re-evaluated after 4 h. In this study, EN interruption was defined as any cessation of enteral feeding lasting ≥4 h, either due to feeding intolerance or due to procedure-related suspension (e.g., extubation, cannula repositioning, imaging). Persistent intolerance for 3–5 days triggered the initiation of parenteral nutrition (PN) as a supplement. These assessments and adjustments were jointly conducted by the attending physician and clinical nutritionist, following the unit’s standardized protocol.

None of the patients received immune enhancers, and all EN was administered via a nasogastric tube. Nutritional intake adequacy was defined as the ratio of actual intake to target intake. EN energy deficiency and non-deficiency were classified based on whether the average daily EN intake during the first 5 days of ECMO treatment reached 30% of the target energy (12, 13). Currently, there is no standardized definition for early EN, but most recommendations suggest that initiating EN within 24 to 48 h after PICU admission qualifies as early EN (9, 11). Therefore, in this study, early EN is defined as EN initiated within 48 h of the start of ECMO treatment.

### 2.5 Scoring systems and nutritional assessment

Vasoactive-Inotropic Score is a tool used to quantify and compare the need for vasoactive-inotropic drugs in pediatric populations (14). The main vasoactive-inotropic drugs included are dopamine, dobutamine, epinephrine, norepinephrine, milrinone, and vasopressin. The formula for calculating the VIS is as follows:

VIS = dopamine [μg/(kg⋅min)] + dobutamine [μg/(kg⋅min)] + 10 × milrinone [μg/(kg⋅min)] + 100 × epinephrine [μg/(kg⋅min)] + 100 × norepinephrine [μg/(kg⋅min)] + 10,000 × vasopressin [U/(kg⋅min)] (15).

The PRISM III score (16) was assessed within the first 24 h of ECMO treatment.

This study used the weight-for-age z-score to determine the nutritional status of all pediatric patients upon admission. The weight-for-age z-score was calculated using the online tool available at https://reference.medscape.com/guide/medical-calculators.

### 2.6 Statistical analysis

Data analysis was performed using SPSS version 27.0. Continuous variables with a normal distribution were presented as mean ± standard deviation (M ± SD), while non-normally distributed variables were described as median (interquartile range) (M, Q1, Q3). The *t*-test or Mann-Whitney U test was used to compare means and medians between groups. Categorical variables were presented as counts (%) and compared using the chi-square test (χ^2^) or Fisher’s exact test. Spearman correlation analysis and effect size analysis were conducted to explore the associations between VIS, other variables, and the adequacy of energy intake during ECMO. We trained a linear-kernel SVM to predict binary energy adequacy (≥ 30% vs < 30%) using fivefold cross-validation. Model performance was evaluated by the area under the receiver operating characteristic curve (AUC) and classification accuracy across folds. Feature importance was estimated via permutation importance and normalized to sum to 100% across predictors. A *p*-value of less than 0.05 was considered statistically significant.

## 3 Results

### 3.1 Study flow

**FIGURE 1:**
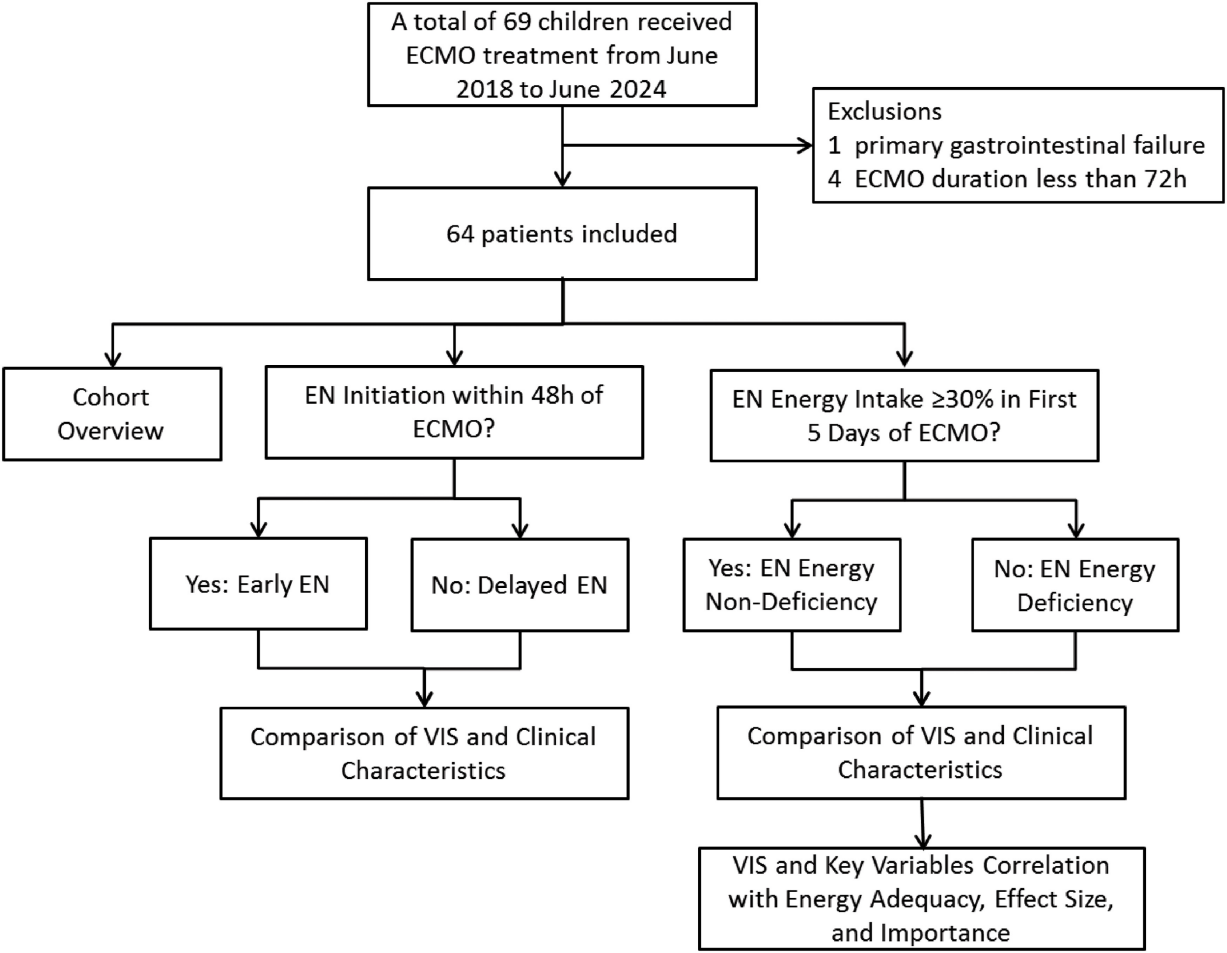
Flowchart of patient screening and grouping (ECMO, extracorporeal membrane oxygenation; EN, enteral nutrition; VIS, vasoactive-inotropic score).

During the study period, a total of 69 pediatric patients received ECMO treatment. Ultimately, 64 patients who met the inclusion criteria were included in the final analysis. Figure 1 shows the study flow and the reasons for exclusion of certain participants.

### 3.2 Overview of the study cohort

The majority of pediatric patients received vasoactive-inotropic drugs during the first three days of ECMO, with the VIS peaking on the first day of ECMO treatment. A total of 43 patients successfully received early EN, and 34% of patients achieved an average EN energy intake adequacy of ≥30%. Ultimately, 67% of the patients survived to discharge. The demographic data of the 64 ECMO pediatric patients, ECMO treatment details, EN intake, and VIS are summarized in Table 1.

**TABLE 1:** Demographic and clinical characteristics of the entire cohort undergoing ECMO treatment.

Variables	Entire Cohort (*n* = 64)
Age (months), M (Q1, Q3)	39.00 (6.21, 106.00)
Male, n (%)	37 (57.81%)
Weight-for-age Z-score, M (Q1, Q3)	−0.44 (−1.36, 0.42)
Total ECMO duration (hours), M (Q1, Q3)	121.20 (96.00, 216.60)
V-A mode, *n* (%)	54 (84.38%)
Maximum ECMO flow [ml/kg/min], M (Q1, Q3)	72.50 (23.40, 96.25)
ECMO for severe pneumonia/ARDS, *n* (%)	39 (60.94%)
PRISM III score, M ± SD	15.11 ± 6.80
CRRT during ECMO, *n* (%)	36 (56.25%)
EN initiated within 48 h, *n* (%)	43 (67.19%)
Average of the first 5 days of ECMO	
Energy intake (kcal/kg), M (Q1, Q3)	9.85 (3.65, 19.73)
Energy adequacy, M (Q1, Q3)	0.17 (0.00, 0.32)
Protein intake (g/kg), M (Q1, Q3)	0.26 (0.06, 0.53)
Protein adequacy, M (Q1, Q3)	0.16 (0.03, 0.35)
Energy intake adequacy ≥ 30%, *n* (%)	22 (34.38%)
Interruptions (times/day), M (Q1, Q3)	0.78 (0.45, 1.02)
VIS in first 5 days of ECMO, M (Q1, Q3)	
VIS on day 1	15.00 (0.93, 31.25)
VIS on day 2	10.00 (0.00, 19.25)
VIS on day 3	7.40 (0.00, 14.77)
VIS on day 4	0.00 (0.00, 6.98)
VIS on day 5	0.00 (0.00, 5.00)
Maximum VIS	19.83 (11.78, 39.50)
Pre-ECMO VIS	5.00 (0.00, 18.02)
Successful ECMO weaning, *n* (%)	50 (78.12%)
IFD, M (Q1, Q3)	7.00 (0.00, 13.00)
Total mechanical ventilation (h), M (Q1, Q3)	249.75 (173.75, 428.10)
Total hospital LOS (days), M (Q1, Q3)	30 (23, 50)
Survived to discharge, *n* (%)	43 (67.19%)

ECMO, extracorporeal membrane oxygenation; V-A mode, veno-arterial mode; ARDS, acute respiratory distress syndrome; PRISM III, pediatric risk of mortality score, version 3; CRRT, continuous renal replacement therapy; EN, enteral nutrition; VIS, vasoactive-inotropic score; IFD, 28-day PICU-free days; LOS, length of Stay. Quantitative data are presented as median (interquartile range), and categorical data as count (percentage).

Of note, among the indications for ECMO, respiratory causes accounted for the majority (61%), while the remaining causes included post-cardiac surgery low cardiac output syndrome (*n* = 9), fulminant myocarditis (*n* = 6), refractory septic shock (*n* = 7), congenital diaphragmatic hernia (*n* = 2), and ECPR after cardiac arrest (*n* = 1). Neonates comprised 9% of the cohort. All 10 V-V ECMO patients were older children with severe pneumonia/ARDS (median age 119 months), whereas V-A ECMO patients were younger (median age 18.5 months).

### 3.3 VIS and clinical characteristics and outcomes in the early vs. delayed EN groups

A total of 43 pediatric patients (67.2%) successfully initiated EN within 48 h of ECMO initiation, with 33 patients (51.6%) starting EN within 24 h. Based on whether EN was effectively initiated within 48 h, patients were divided into the early EN group and the delayed EN group. There were no significant statistical differences in VIS over the first few days between the two groups. The majority of patients in the early EN group had severe pneumonia or ARDS. The delayed EN group had slightly more severe conditions, with a significantly higher number of EN interruptions and lower protein and energy intake compared to the early EN group (*P* < 0.05) (Table 2).

**TABLE 2:** Comparison of key characteristics and outcomes between early EN and delayed EN groups.

Variables	Early EN (*n* = 43)	Delayed EN (*n* = 21)	U/Z/χ ^2^	P
Age (months), M (Q1, Q3)	30.0 (9.5, 106.0)	49.0 (4.0, 106.0)	456.5	0.949
Male, *n* (%)	20 (46.5%)	7 (33.3%)	0.536	0.464
Weight-for-age Z-score, M (Q1, Q3)	−0.7 (−1.6, 0.4)	−0.2 (−1.2, 0.5)	388.0	0.368
Total ECMO duration (h), M (Q1, Q3)	129.6 (111.6, 200.4)	115.2 (86.4, 264.0)	531.5	0.255
V-A mode, *n* (%)	36 (83.7%)	18 (85.7%)	0.04	0.841
Maximum ECMO flow (ml/kg/min), M (Q1, Q3)	70.5 (0.0, 93.4)	76.9 (58.0, 100.0)	418	0.634
ECMO for severe pneumonia/ARDS, *n* (%)	32 (74.4%)	7 (33.3%)	8.35	0.004
PRISM III score, M (Q1, Q3)	16.0 (10.0, 20.5)	22.0 (14.0, 24.0)	305	0.037
CRRT during ECMO, *n* (%)	23 (53.5%)	13 (61.9%)	0.372	0.541
Average of the first 5 days of ECMO				
Caloric intake (kcal/kg), M (Q1, Q3)	13.40 (8.29, 21.84)	2.80 (0.00, 6.30)	772.5	<0.001
Protein intake (g/kg), M (Q1, Q3)	0.41 (0.20, 0.60)	0.02 (0.00, 0.06)	685.2	<0.001
Energy intake adequacy ≥ 30%, *n* (%)	20 (46.5%)	2 (9.6%)	8.12	0.004
Interruptions, (times/day)	0.66 (0.44, 1.02)	1.02 (0.87, 1.25)	270.0	0.028
VIS in first 5 days of ECMO, M (Q1, Q3)				
Day 1 VIS	11.0 (0.3, 26.5)	20.0 (10.0, 32.0)	360	0.190
Day 2 VIS	7.5 (0.0, 17.0)	18.0 (0.0, 23.4)	342	0.114
Day 3 VIS	7.0 (0.0, 12.0)	12.2 (0.0, 19.4)	331.5	0.084
Day 4 VIS	2.0 (0.0, 7.0)	0.0 (0.0, 6.0)	474	0.733
Day 5 VIS	0.0 (0.0, 6.8)	0.0 (0.0, 0.0)	546	0.102
Maximum VIS	15.5 (10.3, 33.9)	30.0 (19.4, 42.0)	300.5	0.031
Pre-ECMO VIS	5.0 (0.0, 17.9)	0.0 (0.0, 17.4)	522	0.298
Successful ECMO weaning, *n* (%)	35 (81.4%)	15 (71.4%)	0.34	0.559
IFD, M (Q1, Q3)	7.0 (0.0, 12.5)	3.0 (0.0, 14.0)	479.5	0.686
VFD, M (Q1, Q3)	16.0 (9.2, 19.9)	15.6 (0.0, 20.3)	496	0.527
Total hospital LOS, M (Q1, Q3)	30.0 (23.0, 48.5)	28.0 (19.0, 63.0)	450	0.989
Survived to discharge, *n* (%)	29 (67.4%)	14 (66.7%)	0.825	0.986

ECMO, extracorporeal membrane oxygenation; V-A mode, veno-arterial mode; ARDS, acute respiratory distress syndrome; PRISM III, pediatric risk of mortality score, version 3; CRRT, continuous renal replacement therapy; EN, enteral nutrition; VIS, vasoactive-inotropic score; IFD, 28-day PICU-free days; VFD, ventilator-free days in 28 days; LOS, length of stay. Quantitative data are presented as median (interquartile range), and categorical data as count (percentage).

### 3.4 Clinical characteristics and outcomes of the EN energy deficiency and non-deficiency groups

**FIGURE 2:**
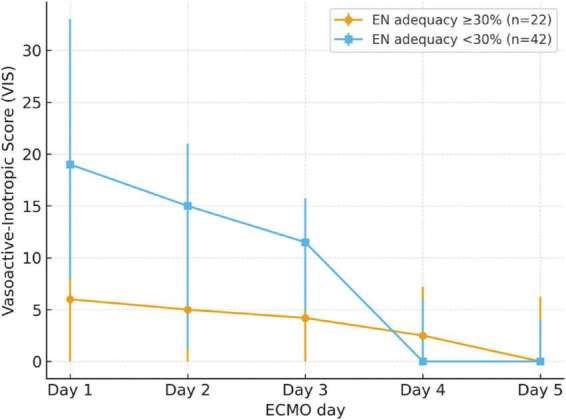
Trend of VIS over the first 5 days of ECMO, stratified by EN energy adequacy groups. Patients with EN energy adequacy <30% (deficient group, *n* = 42) had consistently higher VIS during days 1–3 compared with those with ≥30% adequacy (non-deficient group, *n* = 22), with convergence thereafter. Error bars represent interquartile ranges.

Pediatric patients were divided into the non-deficiency group (≥ 30%) and the EN energy deficiency group (< 30%) based on their energy intake from EN over the first 5 days. A total of 42 patients (65.6%) were classified as having EN energy deficiency (< 30%). The EN energy deficiency group had significantly higher VIS on day 1, day 2, day 3, and maximum VIS (*P* < 0.05), with significantly lower energy and protein intake, as well as a higher number of EN interruptions compared to the non-deficiency group (*P* < 0.05). See Table 3. These trends are illustrated in Figure 2, which shows that patients in the EN energy deficient group had consistently higher VIS during the first three days of ECMO, with convergence thereafter.

**TABLE 3:** Comparison of key characteristics and outcomes between EN energy non-deficiency and deficiency groups.

Variables	EN Energy non-deficiency (≥ 30%) (*n* = 22)	EN Energy deficiency (< 30%) (*n* = 42)	U/Z/χ ^2^	P
Age (months), M (Q1, Q3)	64.0 (12.43, 100.75)	29.00 (4.31, 106.00)	543.0	0.26
Male, *n* (%)	11 (50.00%)	26 (61.90%)	0.83	0.52
Weight-for-age Z-score, M (Q1, Q3)	−0.56 (−1.18, 0.08)	−0.35 (−1.45, 0.49)	449.5	0.87
Total ECMO duration (h), M (Q1, Q3)	115.2 (96.0, 138.0)	147.6 (99.6, 287.4)	316.0	0.04
V-A mode, *n* (%)	19 (86.36%)	35 (83.3%)		0.964
Maximum ECMO flow (ml/kg/min), M (Q1, Q3)	70.8 (0.0, 89.3)	75.9 (55.0, 100.0)	393.0	0.33
ECMO for severe pneumonia/ARDS, *n* (%)	17 (77.27%)	22 (52.38%)	2.79	0.10
PRISM III score, M (Q1, Q3)	12.5 (9.25, 17.75)	20.0 (13.25, 23.75)	−2.84	0.006
CRRT during ECMO, *n* (%)	8 (36.36%)	28 (66.67%)	5.23	0.02
Average of the first 5 days of ECMO				
Caloric intake (kcal/kg), M (Q1, Q3)	21.84 (19.24, 26.86)	7.32 (1.37, 10.12)	825.0	<0.001
Protein intake (g/kg), M (Q1, Q3)	0.60 (0.52, 0.79)	0.15 (0.02, 0.29)	806.0	<0.001
EN within 48 h, yes	20 (90.91%)	23 (54.76%)	8.312	0.004
Interruptions, (times/day)	0.45 (0.25, 0.71)	0.96 (0.54, 1.03)	472.0	0.004
VIS in first 5 days of ECMO, M (Q1, Q3)				
Day 1 VIS	6.00 (0.00, 18.62)	19.00 (8.00, 33.00)	291.0	0.015
Day 2 VIS	5.00 (0.00, 10.15)	15.00 (1.25, 21.00)	301.0	0.021
Day 3 VIS	4.19 (0.00, 7.83)	11.50 (4.94, 15.75)	296.0	0.018
Day 4 VIS	2.50 (0.00, 7.21)	0.00 (0.00, 6.00)	517.0	0.40
Day 5 VIS	0.00 (0.00, 6.23)	0.00 (0.00, 3.93)	483.5	0.72
Maximum VIS	13.06 (7.21, 19.92)	22.60 (15.25, 48.75)	266.0	0.006
Pre-ECMO VIS	5.00 (0.00, 10.12)	5.00 (0.00, 22.92)	453.0	0.90
Successful ECMO weaning, *n* (%)	20 (90.91%)	30 (71.43%)	0.78	0.11
IFD, M (Q1, Q3)	8.0 (1.75, 13.75)	4.5 (0.0, 13.0)	551.5	0.20
VFD, M (Q1, Q3)	15.15 (10.15, 18.15)	15.95 (0.625, 20.38)	439.5	0.75
Total hospital LOS, M (Q1, Q3)	34.0 (27.75, 50.0)	27.5 (22.0, 41.0)	577.5	0.10
Survived to discharge, *n* (%)	18 (81.82%)	25 (59.52%)	0.68	0.10

ECMO, extracorporeal membrane oxygenation; V-A mode, veno-arterial mode; ARDS, acute respiratory distress syndrome; PRISM III, pediatric risk of mortality score, version 3; CRRT, continuous renal replacement therapy; EN, enteral nutrition; VIS, vasoactive-inotropic score; IFD, 28-day PICU-free days; VFD, ventilator-free days in 28 days; LOS, length of stay. Quantitative data are presented as median (interquartile range), and categorical data as count (percentage).

### 3.5 Analysis of predictive indicators for energy intake status in the early phase of ECMO

To identify the main factors influencing EN energy intake adequacy, including VIS during the first three days of ECMO, number of EN interruptions, PRISM III score, and the use of CRRT, we conducted Spearman correlation analysis, effect size analysis (Cohen’s d), and a machine learning model (Support Vector Machine, SVM) for evaluation.

The VIS during the first three days of ECMO showed a moderate negative correlation with EN energy intake adequacy (*P* < 0.05) and had a significant effect size between the energy adequacy groups (Cohen’s d = −0.42, −0.59, and −0.56 for days 1, 2, and 3, respectively). SVM model validation further demonstrated that VIS on day 1 and day 2 contributed the most to predicting energy intake status, with importance scores of 25.7% and 27.4%, respectively. The PRISM III score showed a moderate effect size (Cohen’s d = −0.45). The number of EN interruptions had an importance score of 18.9% in the SVM model but was still lower than the VIS on the first two days (Table 4).

**TABLE 4:** Predictive indicators for enteral energy intake adequacy during the early phase of ECMO.

Variables	Spearman correlation coefficient (rho)	*P*-value (Spearman)	Effect Size (Cohen’s d orΦ)	SVM importance
Day 1 VIS	−0.34	0.016	Cohen’s *d* = −0.42	25.7%
Day 2 VIS	−0.30	0.022	Cohen’s *d* = −0.59	27.4%
Day 3 VIS	−0.31	0.018	Cohen’s *d* = −0.56	4.7%
EN interruptions	−0.23	0.048	Cohen’s *d* = −0.21	18.9%
PRISM III score	−0.19	0.079	Cohen’s *d* = −0.45	16.7%
CRRT (Yes/No)	0.14	0.115	Φ = 0.29	6.7%

The Spearman correlation coefficient represents the relationship between a variable and energy intake adequacy. An absolute value of ≥0.3 indicates a moderate or stronger correlation, which has practical significance. A *P*-value < 0.05 indicates that the correlation is statistically significant and not due to chance. Cohen’s d or Phi (Φ) coefficient indicates the effect size of the difference between groups in terms of energy intake adequacy, with an absolute value of Cohen’s *d* ≥ 0.5 or Φ ≥ 0.30 considered a medium or greater effect. SVM importance reflects each variable’s relative contribution to predicting energy adequacy; higher percentages indicate greater contribution. VIS on days 1 and 2 had the highest importance and were the strongest predictors. The SVM model was evaluated using fivefold cross-validation, showing good accuracy and stability.

## 4 Discussion

The application of early EN in ECMO pediatric patients with hemodynamic instability remains under investigation, and there is currently limited evidence regarding the association between the use of vasoactive-inotropic drugs and EN intake in pediatric ECMO patients. In this study of 64 pediatric ECMO patients, the use of vasoactive-inotropic drugs did not significantly impede the initiation of EN. However, higher VIS values during the first three days of ECMO were associated with lower adequacy of EN energy intake, particularly on days 1 and 2, which emerged as effective predictors of energy intake sufficiency. These findings provide valuable insights for clinicians in making nutritional therapy decisions for pediatric ECMO patients and offer important information for prognosis consultations with families.

Nutritional support is crucial for the treatment of critically ill pediatric patients (17). Among children receiving ECMO support, the adequacy of EN is generally low (18), and sufficient EN energy intake is closely associated with lower mortality rates in ECMO patients (19). Early EN has been shown to be feasible and is associated with better clinical outcomes (20, 21). The Extracorporeal Life Support Organization recommends initiating EN as early as possible when the gastrointestinal tract is functional, noting that adverse reactions to EN during the use of vasoactive-inotropic drugs are generally acceptable (9). However, due to the limited number of pediatric ECMO patients, related research is relatively insufficient, and the evidence level in the relevant guidelines is low. Therefore, there remains controversy surrounding the optimal nutritional support strategies for critically ill children requiring ECMO rescue support.

Whether pediatric ECMO patients can tolerate earlier EN and the specific impact of vasoactive-inotropic drugs on the timing of EN initiation have not been clearly addressed in the guidelines. Since the majority of pediatric ECMO patients are those with respiratory and circulatory failure, this study found that more than 80% of them required vasoactive-inotropic drug support during the early phase of ECMO. Although the literature reports that vasoactive-inotropic drugs can lead to insufficient intestinal perfusion (22), severe gastrointestinal adverse reactions were rare among ECMO patients receiving both vasoactive-inotropic drugs and EN in this study. Only one patient developed gastrointestinal stress ulcers after four days of ECMO treatment, and another one developed necrotizing enterocolitis after 1 week. The patient with necrotizing enterocolitis eventually died, while the patient with stress ulcers survived. However, it could not be definitively established whether these conditions were directly related to the use of EN and vasoactive-inotropic drugs.

The results of this study show that the use of vasoactive-inotropic drugs did not significantly impede the initiation of early EN in pediatric ECMO patients. This is consistent with existing guidelines, which recommend initiating EN as early as possible after the patient’s clinical condition stabilizes, without excessive concern about the use of vasoactive-inotropic drugs (9). VIS has been validated as a tool for assessing the acute phase of pediatric critical illness and has been shown to strongly predict poor outcomes (23). However, our study further indicates that although the VIS in the delayed EN group was higher than that in the early EN group during the first three days of ECMO, the difference was not statistically significant, indicating similar hemodynamic support between groups and a largely balanced hemodynamic status.

Although VIS did not have a significant impact on the early initiation of EN, children in the delayed EN group had significantly lower energy and protein intake from EN, and higher times of EN interruptions. Additionally, the PRISM III scores in the delayed EN group were higher than those in the early EN group, indicating more severe illness in these patients. This may be one of the potential reasons for the failure to initiate EN early and may have increased the likelihood of EN interruptions, thereby affecting the adequacy of energy and protein intake as well as EN tolerance.

In the EN energy deficiency group, the VIS scores during the first three days of ECMO treatment, as well as the maximum VIS, were significantly higher than in the non-deficiency group. This may be due to the more unstable hemodynamic status in the former group, requiring the use of more vasoactive-inotropic drugs, which affected gastrointestinal function and led to insufficient energy intake in the early phase of ECMO. However, by the fourth and fifth days of ECMO treatment, the differences in VIS scores between the two groups were no longer significant, indicating that hemodynamic stability gradually improved over time, and the conditions in both groups became more comparable.

In the analysis of predictive indicators for energy intake status, we found that VIS levels during the first three days were significantly negatively correlated with insufficient energy intake, and VIS had a strong effect on differences in energy intake adequacy (Cohen’s d = −0.42, −0.59, and −0.56, *P* < 0.05). The machine learning SVM model showed that VIS on day 1 and day 2 were the most important variables for predicting energy intake status, contributing significantly to whether patients achieved adequate energy intake (25.7% and 27.4%, respectively). This indicates that VIS levels during the first two days of ECMO directly affect patients’ nutritional status and are important predictive indicators of energy intake adequacy. These findings suggest that clinicians should pay close attention to daily VIS, especially during the first two days, and adjust nutritional strategies or optimize hemodynamic management to improve energy intake adequacy.

Additionally, similar to the findings of a study on nutritional intake in pediatric ECMO patients (20), we also found that the group of patients with poorer energy intake had more EN interruptions and relatively longer ECMO treatment durations, although there was no significant difference in the final discharge survival rates. In our study, EN interruptions were somewhat associated with insufficient energy intake. Although the effect size was small, EN interruptions showed some importance in the SVM model (18.9%), suggesting that more frequent interruptions can further contribute to insufficient EN energy intake. However, they were not the most critical factor in determining overall energy intake and did not severely impact energy intake adequacy.

We suggest that early EN insufficiency in ECMO patients may primarily affect short-term metabolic and recovery processes, leading to a prolonged duration of ECMO. This extension may reflect a slower overall recovery or more complex conditions, but it does not necessarily have a direct impact on final survival outcomes. Ultimately, the most important factor influencing prognosis remains the underlying nature of the disease itself. Initial EN insufficiency may not directly lead to worse survival outcomes, especially if EN gradually recovers later during ECMO with PN supplementation, which could limit its short-term impact on mortality. However, these patients may still face prolonged rehabilitation and increased risks of impaired growth or neurodevelopmental challenges. Future longitudinal studies with extended follow-up are warranted to clarify the impact of early nutritional adequacy during ECMO on recovery trajectories and long-term quality of life.

Although this study provides valuable insights, there are several limitations. First, the number of pediatric patients receiving ECMO treatment was relatively small, and being limited to two PICUs may restrict the external validity and generalizability of the findings. Second, within the scope of this study, certain variables that may influence the initiation of EN and energy intake, such as specific pathophysiological conditions or the use of other medications, were not captured, and because of the limited number of outcome events we did not perform multivariable regression to adjust for additional potential confounders. Third, estimation of energy requirements relied on the Schofield formula, which is widely used but may not accurately reflect true energy expenditure in critically ill children, potentially leading to deviation between actual energy expenditure and the calculated values. Therefore, future multi-center, large-sample prospective studies are needed to further validate these findings. Such studies should also incorporate more precise methods of nutritional assessment and explore a broader range of influencing factors.

## 5 Conclusion

This study indicates that although the use of vasoactive-inotropic drugs did not hinder the initiation of EN, the VIS during the first three days of ECMO is a crucial factor influencing the adequacy of EN energy intake. Clinicians should pay close attention to the use of vasoactive-inotropic drugs during this period in the nutritional management of ECMO patients and adjust nutritional strategies to improve energy intake adequacy, thereby enhancing patient outcomes.

## Data Availability

Publicly available datasets were analyzed in this study. This data can be found here: Dang, Hongxing (2024), “EN in Pediatric ECMO,” Mendeley Data, V1, doi: 10.17632/jcd7nn3mwy.1.
